# A General Design Rule to Manipulate Photocarrier Transport Path in Solar Cells and Its Realization by the Plasmonic-Electrical Effect

**DOI:** 10.1038/srep08525

**Published:** 2015-02-17

**Authors:** Wei E. I. Sha, Hugh L. Zhu, Luzhou Chen, Weng Cho Chew, Wallace C. H. Choy

**Affiliations:** 1 Department of Electrical and Electronic Engineering, The University of Hong Kong, Pokfulam Road, Hong Kong; 2 Department of Electrical and Computer Engineering, University of Illinois, Urbana-Champaign, Illinois 61801, USA

## Abstract

It is well known that transport paths of photocarriers (electrons and holes) before collected by electrodes strongly affect bulk recombination and thus electrical properties of solar cells, including open-circuit voltage and fill factor. For boosting device performance, a general design rule, tailored to arbitrary electron to hole mobility ratio, is proposed to decide the transport paths of photocarriers. Due to a unique ability to localize and concentrate light, plasmonics is explored to manipulate photocarrier transport through spatially redistributing light absorption at the active layer of devices. Without changing the active materials, we conceive a plasmonic-electrical concept, which tunes electrical properties of solar cells via the plasmon-modified optical field distribution, to realize the design rule. Incorporating spectrally and spatially configurable metallic nanostructures, thin-film solar cells are theoretically modelled and experimentally fabricated to validate the design rule and verify the plasmonic-tunable electrical properties. The general design rule, together with the plasmonic-electrical effect, contributes to the evolution of emerging photovoltaics.

Due to a unique ability to localize, guide, and concentrate light at the nanoscale, plasmonics^1^ finds a special functionality to improve optical properties of thin-film photovoltaic devices^2^,^3^,^4^,^5^,^6^. By introducing metallic nanostructures, light absorption at the active layer of devices will be enhanced, which undoubtedly boosts short-circuit current (*J*_sc_) and thus power conversion efficiency (PCE)^6^,^7^,^8^,^9^,^10^,^11^,^12^,^13^,^14^. Currently, a wideband and angle-insensitive light absorption has been achieved by periodic, quasi-periodic and random metallic nanostructures to approach or break the Yablonovitch^15^,^16^,^17^, Lambertian^18^,^19^, and Shockley-Queisser limits^20^,^21^. However, according to semiconductor physics, bulk recombination of photocarriers (electrons and holes), which is different from light absorption only governed by Maxwell's equations, has a strong influence on electrical properties of solar cells including open-circuit voltage (*V*_oc_) and fill factor (FF) and even predominantly determines the PCE. Going beyond the well-known optical enhancement, in this work, plasmonics is explored to control bulk recombination by modifying photocarrier transport path based on a general design rule. Plasmonics, which localizes intense light very close to metallic nanostructures, can spatially redistribute light absorption at the active layer of photovoltaic devices. As a result, the spatial distribution for excitons (or electron-hole pairs) generation can be plasmonically tunable to optimize photocarrier transport path (or bulk recombination) and thus electrical properties of solar cells. The design rule and its realization by the plasmonic-electrical effect can serve as a guideline for designing photovoltaic devices.

## Results

### Proposed general design rule to reduce bulk recombination

According to semiconductor physics, electrical properties of photovoltaic devices (such as FF and *V*_oc_) strongly depend on bulk recombination of photocarriers. Transport path for a photocarrier is the path from the initial site generated to the electrode collected. A short transport path will reduce the recombination and enhance electrical performances of devices. However, the short transport path cannot be achieved for electrons and holes simultaneously because both photocarriers have the same initial sites; and the total length of the electron and hole transport paths is approximately equal to the thickness of active layer, i.e. 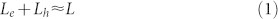
where *L* is the thickness of the active layer. *L_e_* and *L_h_* are the lengths respectively for the electron and hole transport paths.

Besides the transport path, mobility of photocarriers also affect bulk recombination strongly. Driven by the same internal electrostatic field, photocarriers with low mobility will move slowly. Thus low-mobility photocarriers require a long transport time before collected by electrodes. An effective way for reducing recombination loss is to let low-mobility photocarriers travel a short path. In other words, we should force the transport time of electrons and holes almost to be the same. Mathematically, we have 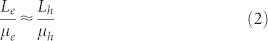
where *μ*_e_ and *μ*_h_ represent mobility of electrons and holes, respectively. The above also suggests electron to hole transport length ratio should be balanced with electron to hole mobility ratio, i.e. *L*_e_/*L*_h_ = *μ*_e_/*μ*_h_. The governing equation (1–2) is a general design rule to decide photocarrier transport path tailored to arbitrary photocarrier mobility.

### Manipulation of photocarrier transport path by plasmonic-electrical effect

**Figure 1:**
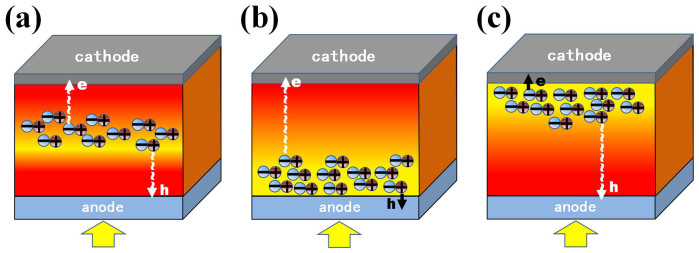
The conceptual diagram of plasmonic-electrical effect. Light absorption is spatially redistributed at the active layer for manipulating transport paths of photocarriers (minus and plus notations denote electrons and holes, respectively). A desired light absorption distribution is realized by spatially tuning the position of metallic NPs with strongly localized plasmonic effects. Light absorption of active materials is optimized by spectrally tuning the shape-dependent plasmonic resonance. The arrows e and h are the transport paths of electrons and holes. Generated electron-hole pairs (excitons) are concentrated (a) at the middle of the active layer, (b) near the anode, and (c) near the cathode, respectively.

Using Eqs. (1) and (2), one could design transport paths or equivalently optimize initial generation sites of excitons. If mobility between electrons and holes are well balanced, the initial sites should be around the middle of active layer. If hole mobility is smaller than electron mobility, excitons should be initially generated near the anode, which guarantees a short transport path of holes. Figure 1 shows a conceptual diagram to manipulate photocarrier generation by embedding configurable metallic nanoparticles (NPs) into the active layer^22^,^23^,^24^. Spatial locations of NPs and spectral locations of plasmonic resonances should be carefully controlled to shorten transport paths of low-mobility photocarriers and maximize light absorption of active materials. A strong and concentrated optical field around NPs will generate dense and inhomogeneous excitons. These non-uniformly distributed excitons not only increase *J*_sc_ but also boost *V*_oc_ and FF through manipulating transport paths of photocarriers to equalize the transport time of electrons and holes. Additionally, light absorption redistribution can be achieved by metallic grating electrodes as well^6^,^19^,^25^,^26^, where the coupling between plasmonic modes and waveguide modes will be manipulated.

### An Approximate Theoretical Model with a Mechanistic Insight

**Figure 2:**
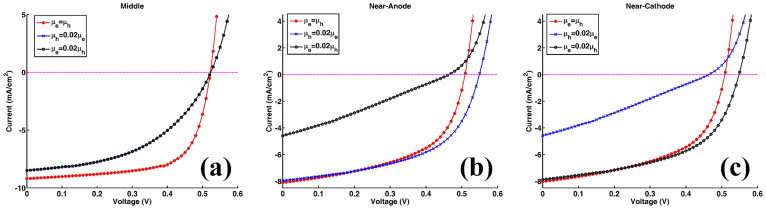
Photocarrier mobility dependence of *J*-*V* characteristics for plasmonic solar cells corresponding to Figures 1(a–c). An approximate theoretical model is adopted for simulating the non-uniform spatial distribution of excitons. Generated electron-hole pairs (excitons) are concentrated (a) at the middle of the active layer, (b) near the anode, and (c) near the cathode, respectively.

To understand the concept that electrical properties of solar cells will be boosted by spatially redistributing light absorption at the active layer, we theoretically study electrical responses of thin-film organic solar cells (OSCs) by using the multiphysics model^27^,^28^,^29^,^30^,^31^,^32^,^33^,^34^,^35^ developed in literatures (also see Theoretical Method Section). Particularly, OSCs with polymer and small-molecule materials always have unbalanced photocarrier mobility, which depend on material morphology, temperature, and experimental procedures (thermal annealing, etc)^26^,^36^. Mobility-dependent current density-voltage (*J*-*V*) responses are investigated with different light absorption (exciton generation) distributions, which can be realized by versatile plasmonic solar cell configurations (see Figure 1). The *J*-*V* curves and corresponding characteristic parameters are presented in Figure 2 and Table 1, respectively. The active layer thickness is *L* = 90 nm. *μ*_e_ = *μ*_h_ = 3 × 10^−3^ cm^2^/(V·s) are set for the case of balanced mobility. Regarding unbalanced cases, hole (electron) mobility is reduced to 1/50 of electron (hole) mobility. The injection barriers are set to be zero for both electrodes. To model the localized plasmonic effect and understand the plasmonic-electrical effect, spatial distributions of exciton generation inside the active layer are approximately set as follows 
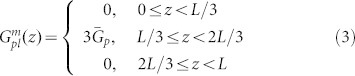

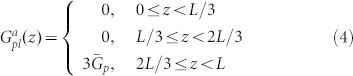

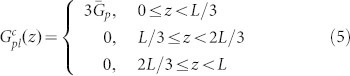
where 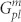, 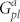, and 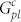 are the exciton generation rates with respect to the three plasmonic solar cells (middle, near-anode, and near-cathode cases) as depicted in Figures 1(a), (b), and (c), respectively. The cathode and anode are located at *z* = 0 and *z* = *L*, respectively. Here all the devices have the same averaged exciton generation rate 
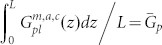
. Physically, light absorption at the active layer is spatially redistributed by the localized plasmonic effect.

From the theoretical results (Tables 1–2 and Figure 2), we find that:

- The spatial distribution of exciton generation has a pronounced influence on electrical responses of OSCs. The mismatch between exciton distribution (controlled by the spatial location of metallic NPs) and photocarrier mobility will lead to poor photovoltaic devices, such as the near-anode case with low-mobility electrons and the near-cathode case with low-mobility holes.
- The best electrical performance (e.g. PCE) can be achieved when electron and hole mobility are well balanced and meanwhile light absorption is concentrated at the middle region of active layer (as listed in the first line of Table 1). For instance, a (standard) planar solar cell cannot achieve best efficiency even if mobility between electron and hole are well balanced. It is because, typically, light intensity is exponentially decayed from the transparent electrode rather than achieving its peak value at the middle region of active layer. On the other hand, although the intensity peak could be around the center of active layer via some smart optical designs, the solar cell does not gain the best efficiency due to unbalanced electron and hole mobility.
- Shortening the transport path of low-mobility photocarriers improves electrical properties of OSCs (*V*_oc_ and FF), such as the near-anode case with low-mobility holes and the near-cathode case with low-mobility electrons. Taking the planar solar cell as an example again, the cell will be particularly helpful for hole collections if its transparent electrode, where light first penetrates, is the anode. This is also the reason why a normal structure of planar OSCs [as shown in Figure 1(b)] has a better performance than an inverted structure when holes have low mobility^33^,^37^.

Comparatively studying Table 2 and Table 1, bulk recombination dominates device performance. Under *V*_oc_ condition (where the total current is equal to zero), recombination rate is very large and is almost equal to generation rate. Therefore, the recombination rates at “different” *V*_oc_ do not have any significance to device performances. Differently, under *J*_sc_ condition or particularly at the maximum power point, larger recombination leads to lower *J*_sc_ and FF, which strongly affect PCE of devices. The plasmonic-electrical effect, which shortens the transport path of low-mobility photocarriers, indeed reduces bulk recombination and boosts the device efficiency. It should be noted that we adopted a simplified mathematical model for exciton generation rates which are zeros at some regions of active layer. From the intuitive model, one can understand the importance of light absorption redistribution (without any light absorption enhancements) to the solar cell performance.

### A Rigorous Theoretical Model and Validation

The approximate exciton generation model presented in Eqs. (3–5) offers a mechanistic insight for plasmonically induced light redistribution at the active layer. But a rigorous model for calculating exciton generation of a practical solar cell is needed to accurately describe photocarrier transport and validate the proposed design rule. A small-molecule bulk heterojunction solar cell with an active material of copper phthalocyanine (CuPc): Buckminsterfullerene (C_60_) is theoretically investigated. In contrast to other solar cells, a standard optimized small-molecule solar cell has an ultrathin active layer due to large absorption coefficients of small molecules and a short exciton diffusion length^38^,^39^. Hence, by incorporating metallic NPs into active layer, the small-molecule device favors a very strong near-field plasmonic effect. In this work, silver NPs with a size of 10 nm and a spacing distance of 10 nm are embedded into the active layer. Using such small-sized NPs, which have large metal absorption loss, is an unusual design. These small NPs do not significantly enhance the total absorption of active materials; albeit extraordinarily redistribute light absorption at the active layer^40^. Exciton generation is calculated by the finite-difference frequency-domain method^41^. Regarding optical simulation details, please see Supplementary Note 1. It should be noted that metal absorption by silver NPs, which does not contribute to exciton generation, has been subtracted from the total absorption of active layer in the calculation^23^.

**Figure 3:**
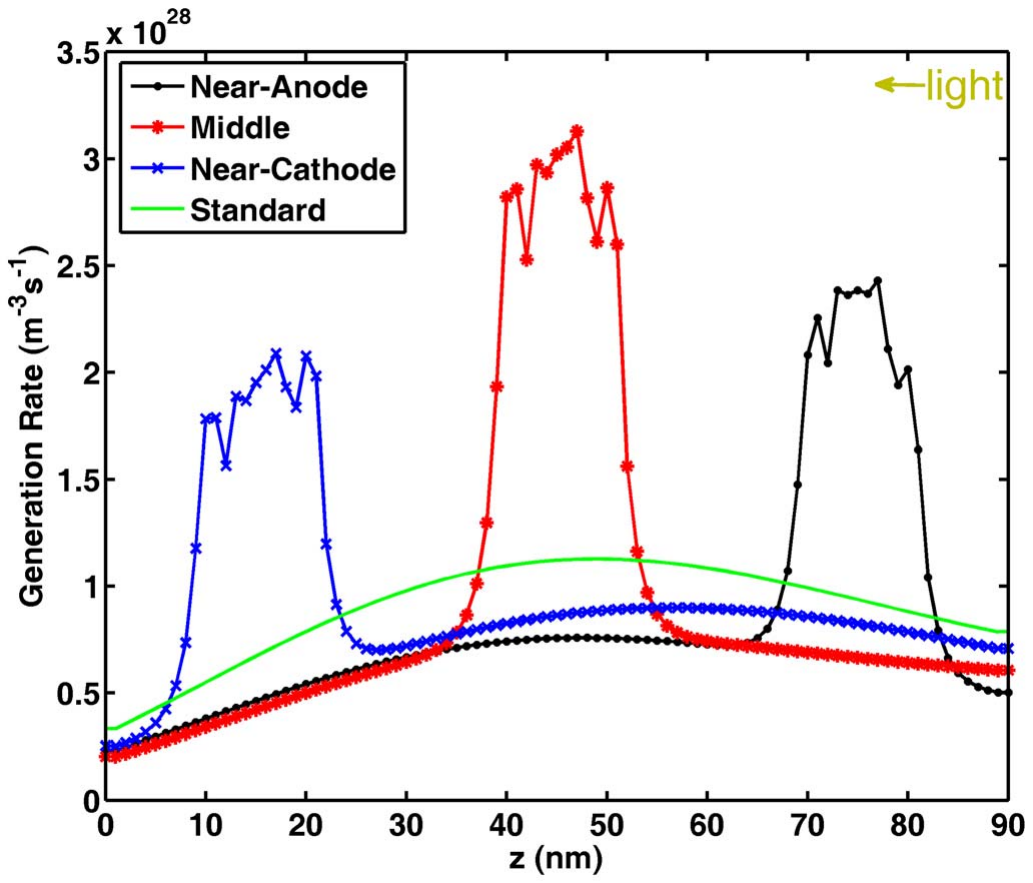
Laterally-averaged exciton generation 
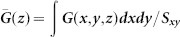
 at the active layer of a small-molecule bulk-heterojunction solar cell. Silver NPs with a size of 10 nm and a spacing distance of 10 nm are embedded into different locations of the active layer corresponding to Figures 1(a–c). The result of a standard cell without NPs is also given for comparisons. The volume-averaged exciton generation 
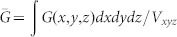
 (10^27^ m^−3^ s^−1^) are 8.34, 9.26, 9.27, 8.93 respectively for the near-anode, middle, near-cathode, and standard cases.

**Figure 4:**
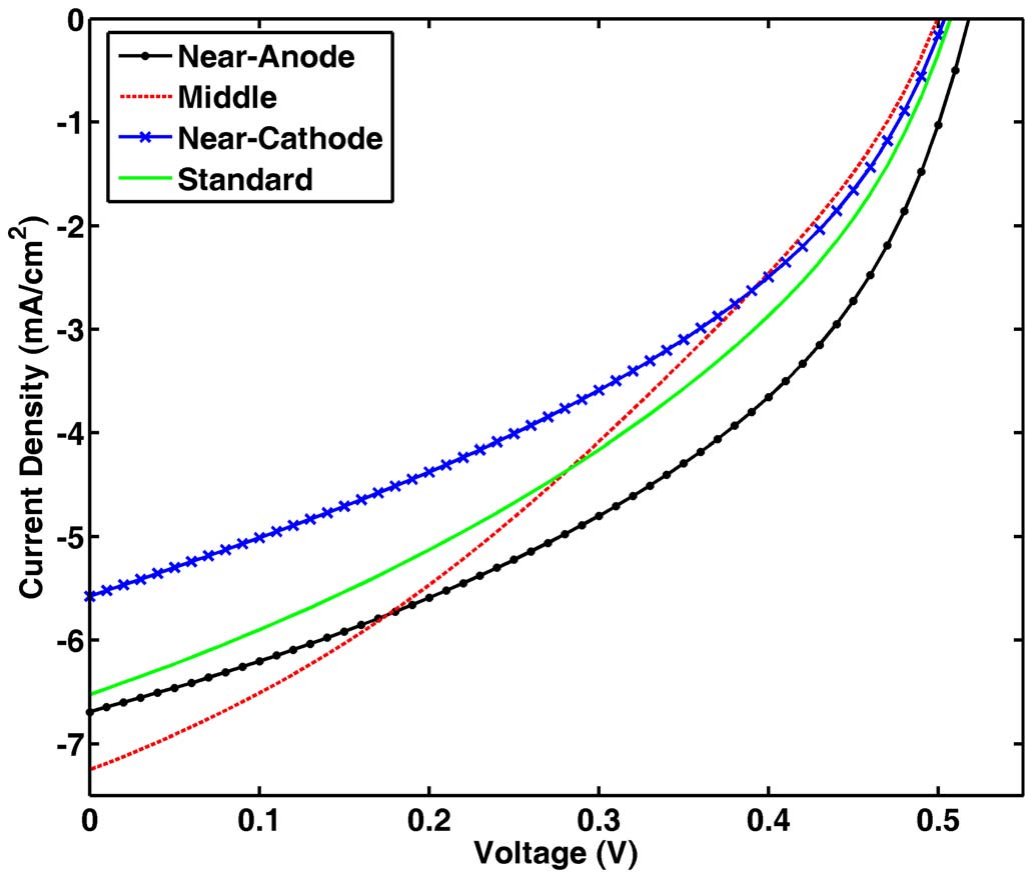
Theoretical *J*-*V* characteristics for plasmonic small-molecule solar cells corresponding to Figures 1(a–c). A rigorous exciton generation result as depicted in Figure 3 is adopted in the simulation. The electron and hole mobility are respectively set to be 10^−2^ cm^2^/(V·s) and 10^−5^ cm^2^/(V·s). The PCE are 1.50%, 1.23%, 1.09%, and 1.26% respectively for the near-anode, middle, near-cathode, and standard cases.

Figure 3 shows the laterally-averaged exciton generation along the vertical direction of active layer. Compared to the standard cell without NPs, plasmonically induced light absorption redistribution and thus non-uniform exciton generation can be clearly observed (also see Figure S2). Hence, the approximate exciton model given by Eq. (4) is quite reasonable. Figure 4 illustrates the *J*-*V* response with respect to the exciton generation of Figure 3. The electron and hole mobility are respectively set to be 10^−2^ cm^2^/(V·s) and 10^−5^ cm^2^/(V·s), which are typical values in small molecule devices^39^. Due to short transport paths of holes, the near-anode case achieves the best FF, *V*_oc_ and PCE among all the cells including the standard one, which agrees with the approximate theoretical results as listed in the Table 1 highlighted by bold. However, in view of light blocking effect by metal NPs, the near-anode case induces the worst light absorption according to the exciton generation result (Figure 3). So these unusually-small NPs efficiently redistribute (not significantly enhance) light absorption at the active layer; and boost PCE by the plasmonic-electrical effect.

From Table 1 and Figure 4, *J*_sc_ results show an interesting feature that the middle case obtains the largest *J*_sc_, although with a low FF. Firstly, under *J*_sc_ condition, the drift current is dominantly larger than the diffusion current; and photocarriers are fast swept out of devices. Consequently, bulk recombination is weak, which can be seen in Table 2. From the theoretical results at Table 1, the middle and near-anode cases have comparable *J*_sc_ in contrast to the near-cathode one. The increased *J*_sc_ for the middle case in comparison with the near-anode case is due to a nonlinear behavior for bimolecular recombination of photocarriers. However, bulk recombination becomes larger and larger as the built-in electrostatic field is reduced. At the maximum power point or under *V*_oc _condition, the diffusion current competes with or even dominates over the drift current. Under this situation, shortening the transport path of low-mobility photocarriers will significantly reduce the bulk recombination and boost electrical properties of devices especially for FF and *V*_oc_. Secondly, exciton generation of a practical plasmonic solar cell is the exciton generation of a standard device without metallic NPs multiplied by the spatial-dependent (plasmonic) enhancement factor, which should be obtained with a rigorous simulation. Regarding practical plasmonic devices, the near-anode case [Figure 1(b)] has the shortest transport path of low-mobility holes but blocks a portion of light due to metal absorption. From the results in Figure 3, the volume-averaged exciton generation of the near-anode case gets the lowest value; and is even lower than that of the standard cell without NPs. This is the reason why the near-anode case induces a lower *J*_sc_ compared to the middle case. On the other hand, the near-cathode case [Figure 1(c)] has the longest hole transport path to the anode, which causes a large recombination loss and thus the lowest *J*_sc_. From above discussions, *J*_sc_ of practical solar cells depends not only on the transport path of photocarriers but also on the magnitude of volume-averaged exciton generation rate.

Although the middle case achieves the largest *J*_sc_, the near-anode case still gains the highest PCE confirmed by the rigorous theoretical results. Therefore, *V*_oc_ and FF, which strongly depend on bulk recombination and are tunable with photocarrier transport paths, play more important roles than *J*_sc_ in determining the overall performance of plasmonic devices.

### Experimental Design and Validation

Very recently, it has been reported that device performance of OSCs is affected by the position of a silver film within the active layer^42^. To understand the underlying physics and experimentally demonstrate the general design rule, we fabricated the small-molecule bulk-heterojunction solar cell with the active material of CuPc:C_60_. Different-sized Ag NPs are embedded into different positions of the active layer by using thermal evaporation at different stages of film formation of the active layer. Experimental details are discussed at the Experimental Section. It is worthy of noting that the plasmonic solar cells are fabricated within a high-vacuum chamber, and thus metal NPs are not passivated. The device structures corresponding to Figures 1(a–c) are given as follows:

ITO/MoO_3_ (10 nm)/CuPc:C_60_ (40 nm)/Ag NPs/CuPc:C_60_ (40 nm)/BCP (10 nm)/Ag

ITO/MoO_3_ (10 nm)/CuPc:C_60_ (20 nm)/Ag NPs/CuPc:C_60_ (60 nm)/BCP (10 nm)/Ag

ITO/MoO_3_ (10 nm)/CuPc:C_60_ (60 nm)/Ag NPs/CuPc:C_60_ (20 nm)/BCP (10 nm)/Ag

**Figure 5:**
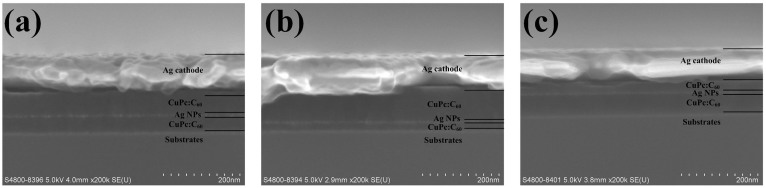
Cross sectional SEM images of small molecule solar cell devices. Ag NPs are embedded (a) at the middle of CuPc:C_60_ active layer, (b) near the anode, and (c) near the cathode, which respectively correspond to Figures 1(a), (b), and (c).

Figure 5 shows the cross sectional scanning electron microscopy (SEM) images of the small molecule solar cells. Ag NPs are well located at a specific region of the CuPc:C_60_ active layer.

**Figure 6:**
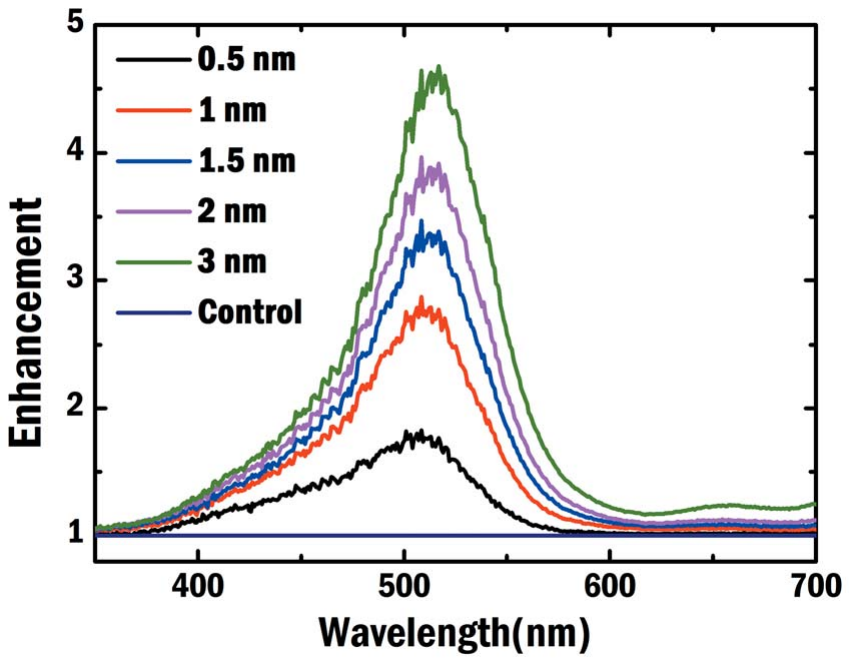
Experimental optical absorption enhancement of CuPc:C_60_ films after embedding Ag NPs. The absorption enhancement of CuPc:C_60_ films embedded with different-sized Ag NPs were measured based on the structure of ITO/CuPc:C_60_ (40 nm)/Ag NPs (various sizes)/CuPc:C_60_ (40 nm). The enhancement is defined as the absorption of plasmonic cells (with Ag NPs) over that of standard cells without Ag NPs. It should be noted that the thickness of initially evaporated metal film as depicted in the legend is not equal to the size of corresponding metal NPs as shown in Figure S3.

To study the plasmonic-electrical effect, on one hand, the size of Ag NPs is tuned to be an optimized value for favoring a strong near-field plasmonic effect. Using the structure of ITO/CuPc:C_60_ (40 nm)/Ag NPs (various sizes)/CuPc:C_60_ (40 nm), the optical absorptance of CuPc:C_60_ films incorporating different-sized Ag NPs (see Figure S3) suggests a strong and tunable plasmonic enhancement (see Figure 6). On the other hand, photocarrier mobility in the bulk-heterojunction active material (CuPc:C_60_) is very unbalanced, which can be confirmed by the experimental results of Figure S4. Here, the hole mobility is four orders of magnitude smaller than the electron one.

**Figure 7:**
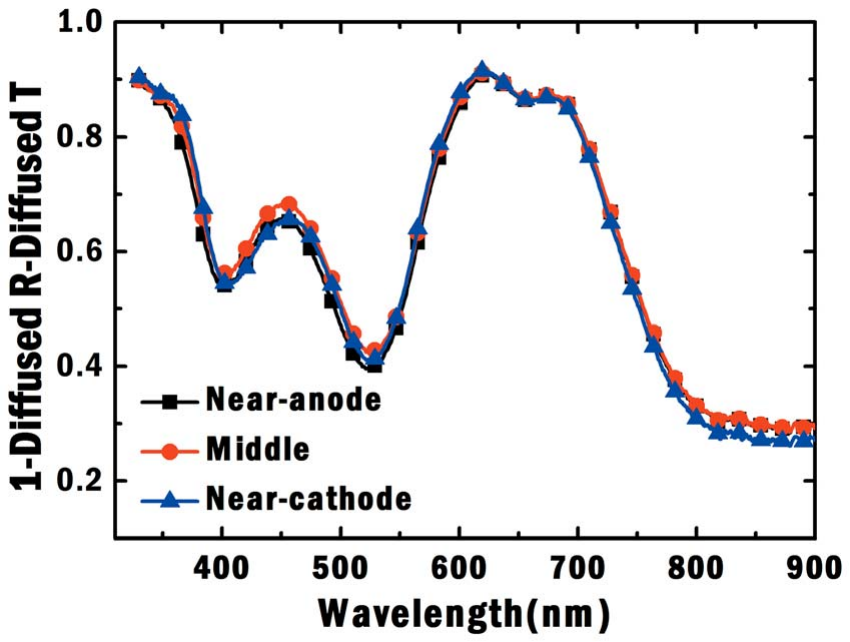
Experimental optical absorption of plasmonic small-molecule solar cells. The optical absorption is obtained by using 1-diffused reflectance (*R*)-diffused transmittance (*T*). Ag NPs are embedded at the active layer with different spatial locations corresponding to Figures 1(a–c). The device structure of the middle case is: ITO/MoO_3_ (10 nm)/CuPc:C_60_ (40 nm)/Ag NPs/CuPc:C_60_ (40 nm)/BCP (10 nm)/Ag; The device structure of the near-anode case is: ITO/MoO_3_ (10 nm)/CuPc:C_60_ (20 nm)/Ag NPs/CuPc:C_60_ (60 nm)/BCP (10 nm)/Ag; The device structure of the near-cathode case is: ITO/MoO_3_ (10 nm)/CuPc:C_60_ (60 nm)/Ag NPs/CuPc:C_60_ (20 nm)/BCP (10 nm)/Ag.

**Figure 8:**
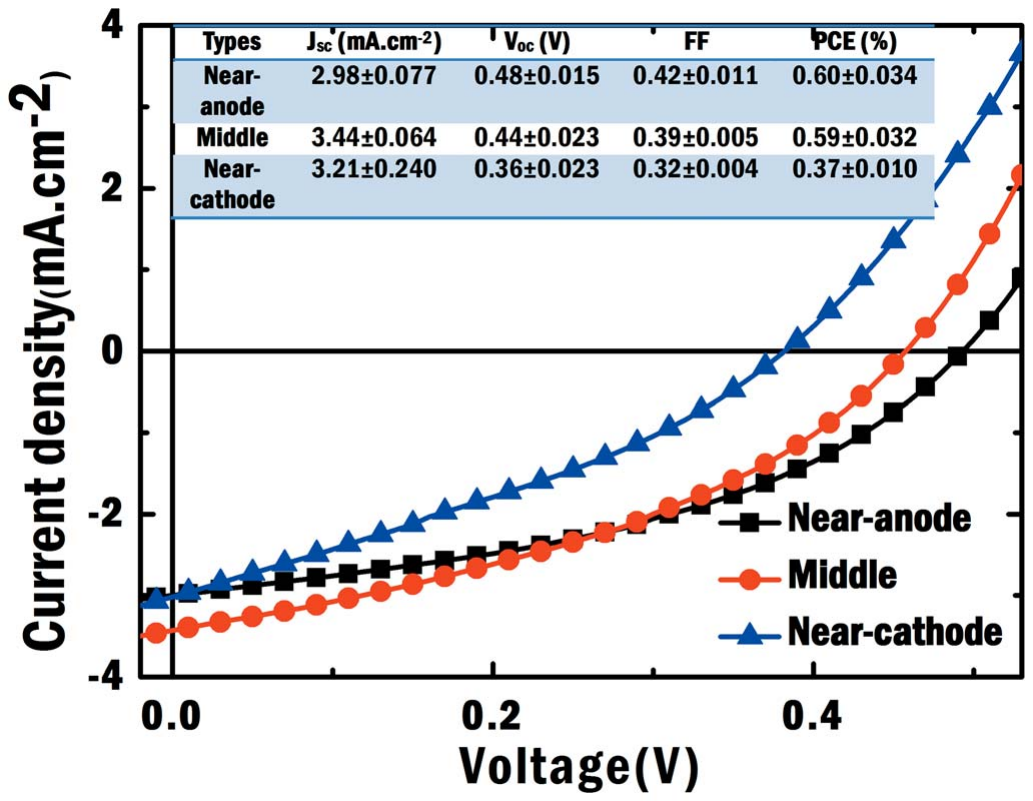
Experimental *J*-*V* characteristics of plasmonic small-molecule solar cells. Ag NPs are embedded at the active layer with different spatial locations corresponding to Figures 1(a–c). The hole mobility is four orders of magnitude smaller than the electron mobility. The statistical parameters of three different plasmonic solar cells are summarized in the inset.

From the experimental results as illustrated in Figures 7 and 8 for different device structures (Figure 5), we find that:

- The optical absorption of plasmonic small molecule solar cells, which is almost independent on the NP positions at the active layer, shows no significant differences (see Figure 7). This agrees with our argument and theoretical results (presented in Figure 3), i.e. light absorption is spatially redistributed by the localized plasmonic effects.
- *V*_oc_ and FF are noticeably improved when Ag NPs are embedded close to the anode (the near-anode case). From experimental results in Figure 8, FF of the near-anode case is 0.42 compared to 0.39 and 0.32 respectively for the middle and near-cathode cases. Moreover, *V*_oc_ of the near-anode case is 0.48 V in contrast to 0.44 V and 0.36 V for the middle and near-cathode cases, respectively. These experimental results agree with the theoretical predictions as listed in the Table 1 (highlighted by bold) and Figure 4. According to Eq. (2) and relevant physical explanations, shortening the transport path of low-mobility holes will reduce bulk recombination resulting in the improved electrical properties.
- *J*_sc_ shows peculiar features after comparing experimental results (Figure 8) to theoretical ones (Figure 4). For the theoretical results, *J*_sc_ of the near-cathode case is much smaller than that of the near-anode case. For the experimental results, *J*_sc_ of the near-cathode case is larger than that of the near-anode case. One of reasons is that bimolecular recombination rate in experimental devices is lower than that in theoretical models based on Langevin theory^43^. Another reason is the exciton quenching at the surface of unpassivated metallic NPs^44^. Thus it is not fair or meaningful to make a direct comparison between the plasmonic devices and standard one. In spite of the peculiar feature of *J*_sc_, the near-anode case still achieves the best efficiency among all the plasmonic devices. Again, *V*_oc_ and FF, highly tunable by the plasmonic-electrical effect, determine the overall plasmonic device performance.

### Extension of the Design Rule to Other Thin-Film Solar Cells

Manipulating photocarrier transport path is not only important to OSCs with low and unbalanced mobility but also important to other thin-film solar cells with high and balance mobility. The amorphous silicon solar cell with a device structure of anode-*p*^+^ silicon (10 nm)-intrinsic silicon (460 nm)-*n*^+^ silicon (30 nm)-cathode is studied. Most photocarriers are generated at the intrinsic layer with high and balanced photocarriers mobility, i.e. *μ*_e_ = 4.6 × 10^−2^ cm^2^/(V·s) and *μ*_h_ = 9.2 × 10^−3^ cm^2^/(V·s). According to our theoretical results (see Supplementary Note 2), the near-anode case, which redistributes light absorption (exciton generation) tailored to electron-to-hole mobility ratio, achieves the best PCE with the maximum *V*_oc_ and FF. Due to a significantly reduced built-in potential at the maximum power point, photocarriers cannot be fast sweep out and thus bulk recombination dominates the device efficiency of solar cells. Additionally, depending on effective mass and average scattering time, mobility of photocarriers, cause different bulk recombination features in different active materials. Consequently, controlling photocarrier transport path by the proposed general design rule is important to thin-film photovoltaics.

In conclusion, we propose a general design rule to manipulate photocarrier transport in photovoltaic devices. Through spatially redistributing light absorption at active layer, the design rule is successfully demonstrated in a configurable plasmonic device with the help of the plasmonic-electrical effect. The dense exciton generation around plasmonic nanostructures provides a flexible and integrated way to both increase *J*_sc_ and simultaneously boost *V*_oc_ and FF. Balancing the transport time for photocarriers with different electron to hole mobility ratio reduces bulk recombination and thus improves the electrical performance of photovoltaic devices. The work is highly important for high-performance photovoltaics.

## Methods

### Device fabrication

The Ag NPs embedded solar cells were fabricated on clean conductive ITO coated glass substrates (sheet resistance of ~20 Ω/sq, purchased from TINWELL Tech. LTD.), which were previously cleaned in ultrasonic bath in detergent, deionized water, acetone, and ethanol, subsequently. After dried by nitrogen gas, substrates were transferred into a vacuum chamber for thermal deposition. MoO_3_ (10 nm, purchased from Aladdin China) was evaporated at the speed of 0.04 nm/sec. A blend CuPc:C_60_ layer (CuPc and C_60_ were purchased from Kintec and Nichem Taiwan, respectively) was deposited at the evaporation rate of 0.05 nm/sec in the organic chamber. Ag NPs were thermally evaporated at different stages of film formation of the active layer. Finally, electron-blocking layer BCP (10 nm, purchased from Kintec) and Ag cathode (Ag pellets were purchased from Kurt J. Lesker) were evaporated in sequence onto the organic active layer to complete the plasmonic solar cells. The evaporation rate and the final thickness of MoO_3_, organic active layer, and Ag NPs were controlled by in situ quartz monitors. All of the materials we utilized in this work were commercially available and used without further purification.

### Characterization

The absorption spectra of Ag NPs embedded organic active films and plasmonic small-molecule solar cells were determined by the diffused reflection (*R*) and transmission (*T*) spectra utilizing a CCD spectrometer (Ocean Optics QE65000) and an integrating sphere. Current-voltage (*J*-*V*) curves of plasmonic small-molecule solar cells were measured using a Keithley 2635 SourceMeter and ABET AM1.5G solar simulator. The morphology of Ag NPs thermally deposited on the surface of organic active layers was characterized by LEO 1530 scanning electron microscope (SEM). Cross sectional SEM images of Ag NPs embedded small molecule solar cells were measured by Hitachi S4800 FEG SEM.

### Theoretical model

The electrical properties of OSCs can be modeled by solving organic semiconductor equations involving Poisson, drift-diffusion and continuity equations^45^,^46^

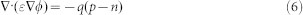

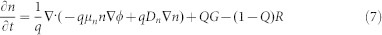

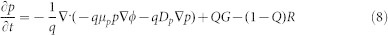
where *q* is the electron charge, *ϕ* is the potential, and *n* and *p* are electron and hole densities, respectively. Moreover, *μ_n_* and *μ_p_* are the mobility of electrons and holes respectively. Furthermore, *D_n_* and *D_p_* are the diffusion coefficients of electrons and holes respectively, which are accessible by the Einstein relations and mobility. *J_n_* = −*qμ_n_n∇ϕ* + *qD_n_∇n* and *J_p_* = −*qμ_p_p∇ϕ* + *qD_p_∇p* are respectively electron and hole current densities, and *G* is the exciton generation rate. Here, the recombination rate *R* is taken as the Langevin bimolecular form^47^; and the field-dependent exciton dissociation probability *Q* is evaluated by the Onsager-Braun theory^48^,^49^.

The potential boundary condition at the electrodes is given by 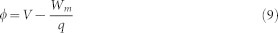
where *V* is the applied bias voltage and *W_m_* is the work function of the electrode. The electrodes are assumed to be ohmic contacts without injection barriers for understanding the influence of photocarrier transport paths on the electrical properties of solar cells, i.e. 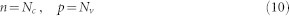
where *N_c_* and *N_v_* are the effective density of states for bulk heterojunction active materials.

## Author Contributions

W.E.I.S. and H.L.Z. contributed equally to the work. W.E.I.S. and L.C. conducted theoretical modeling. H.L.Z. fabricated and measured small-molecule solar cells. W.C.H.C. and W.C.C. planned and supervised the project.

## Supplementary Material

Supplementary Information Supplementary Information

## Figures and Tables

**Table 1:** Electrical parameters for plasmonic solar cells with different photocarrier mobility of active materials and spatial distributions of exciton generation. The *J*_sc_, *V*_oc_, FF, and PCE are the short notations of short-circuit current, open-circuit voltage, fill factor, and power conversion efficiency. N-Anode and N-Cathode are the short notations of near-anode and near-cathode

NPs position	Mobility	*J*_sc _(mA/cm^2^)	FF	*V*_oc_ (V)	PCE (%)
	*μ*_e_ = *μ*_h_	9.198	0.669	0.522	3.21
Middle	***μ*_h_** ** = ** **0.02*μ*_e_**	**8.494**	**0.480**	**0.523**	**2.13**
Fig. 1(a)	*μ*_e_ = 0.02*μ*_h_	8.490	0.480	0.523	2.13
	*μ*_e _ = *μ*_h_	8.099	0.535	0.509	2.21
N-Anode	***μ*_h_ = ** **0.02*μ*_e_**	**7.953**	**0.535**	**0.549**	**2.33**
Fig. 1(b)	*μ*_e_ = 0.02*μ*_h_	4.581	0.278	0.464	0.59
	*μ*_e_ = *μ*_h_	8.022	0.533	0.509	2.18
N-Cathode	***μ*_h_** ** = ** **0.02*μ*_e_**	**4.578**	**0.278**	**0.464**	**0.59**
Fig. 1(c)	*μ*_e_ = 0.02*μ*_h_	7.868	0.533	0.548	2.30

**Table 2:** Averaged bulk recombination rates under short-circuit *J*_sc_, maximum power *P*_max_, and open-circuit *V*_oc_ conditions for plasmonic solar cells with different photocarrier mobility of active materials and spatial distributions of exciton generation. N-Anode and N-Cathode are the short notations of near-anode and near-cathode

NPs position	Mobility	Recombination *J*_sc_ (10^27^ m^−3^s^−1^)	Recombination *P*_max_ (10^27^ m^−3^s^−1^)	Recombination *V*_oc _(10^27^ m^−3^s^−1^)
	*μ*_e_ = *μ*_h_	0.014	0.849	13.507
Middle	*μ*_h_ = 0.02*μ*_e_	0.317	2.792	10.916
Fig. 1(a)	*μ*_e_ = 0.02*μ*_h_	0.317	2.789	10.884
	*μ*_e_ = *μ*_h_	2.762	6.574	15.796
N-Anode	*μ*_h_ = 0.02*μ*_e_	1.678	4.332	12.639
Fig. 1(b)	*μ*_e_ = 0.02*μ*_h_	9.306	11.984	14.759
	*μ*_e_ = *μ*_h_	2.972	6.574	15.700
N-Cathode	*μ*_h_ = 0.02*μ*_e_	8.434	11.036	13.74
Fig. 1(c)	*μ*_e_ = 0.02*μ*_h_	1.574	4.109	12.118
