# Supplementary material for: A General Design Rule to Manipulate Photocarrier Transport Path in Solar Cells and Its Realization by the Plasmonic-Electrical Effect
Source: Sci Rep. 2015 Feb 17;5:8525. doi: 10.1038/srep08525 (PMC4330524; doi:10.1038/srep08525)
Supplement: Supplementary Information [file srep08525-s1.pdf]

A General Design Rule to Manipulate Photocarrier Transport Path in Solar Cells and Its Realization by the Plasmonic-Electrical Effect

Wei E.I. Sha[1], Hugh L. Zhu[1], Luzhou Chen[1], Weng Cho Chew[2], Wallace C.H. Choy[1]
[1] Department of Electrical and Electronic Engineering, The University of Hong Kong, Pokfulam Road, Hong Kong
[2] Department of Electrical and Computer Engineering, University of Illinois, Urbana-Champaign, Illinois 61801, USA
Corresponding Authors: chchoy@eee.hku.hk (Choy); w-chew@uiuc.edu (Chew)

Supporting Information

Supplementary Note 1: theoretical modeling of exciton generation. We employed the finite-difference frequency-domain method [*Opt. Express, 18(6), 5993-6007, 2010*] to calculate the exciton generation for both the standard and plasmonic (nanoparticle-incorporated) small molecule solar cells. The perfectly matched layer absorbing boundary conditions are used at the top and bottom boundaries of the devices. Using the Bloch-Floquet theorem, the periodic boundary conditions are adopted at the lateral boundaries. The complex permittivity of metals (Ag, Au, etc) can be expressed by the Brendel–Bormann model [*Appl. Opt. 37(22), 5271-5283, 1998*]. The complex refractive index of the active material CuPc:C_{60} are measured by ellipsometry as shown in **Figure S1**. The exciton generation rate can be evaluated by

$$G(\mathbf{r}) = \int_{400}^{800} \frac{2\pi}{h} \varepsilon_0 n(\lambda) k(\lambda) \,|\, \mathbf{E}(\mathbf{r},\lambda) \,|^2\, \Gamma(\lambda) d\lambda$$

where n and k are the measured real and imaginary parts of refractive indices of active materials, h is the plank constant, and Γ is the AM 1.5G solar spectrum. **Figure S2** shows exciton generation distribution for the small molecule solar cells with the active layer thickness of 90 nm. Light absorption is redistributed at the active layer after introducing metallic nanoparticles.

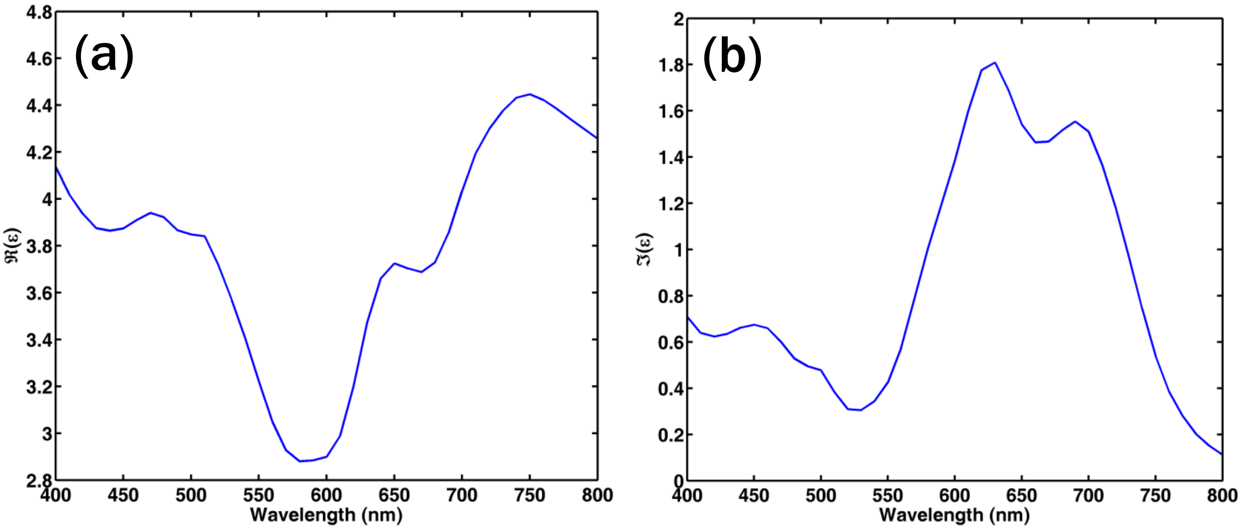

Figure S1. Permittivity of active material CuPc:C_{60}. (a) real part; (b) imaginary part.

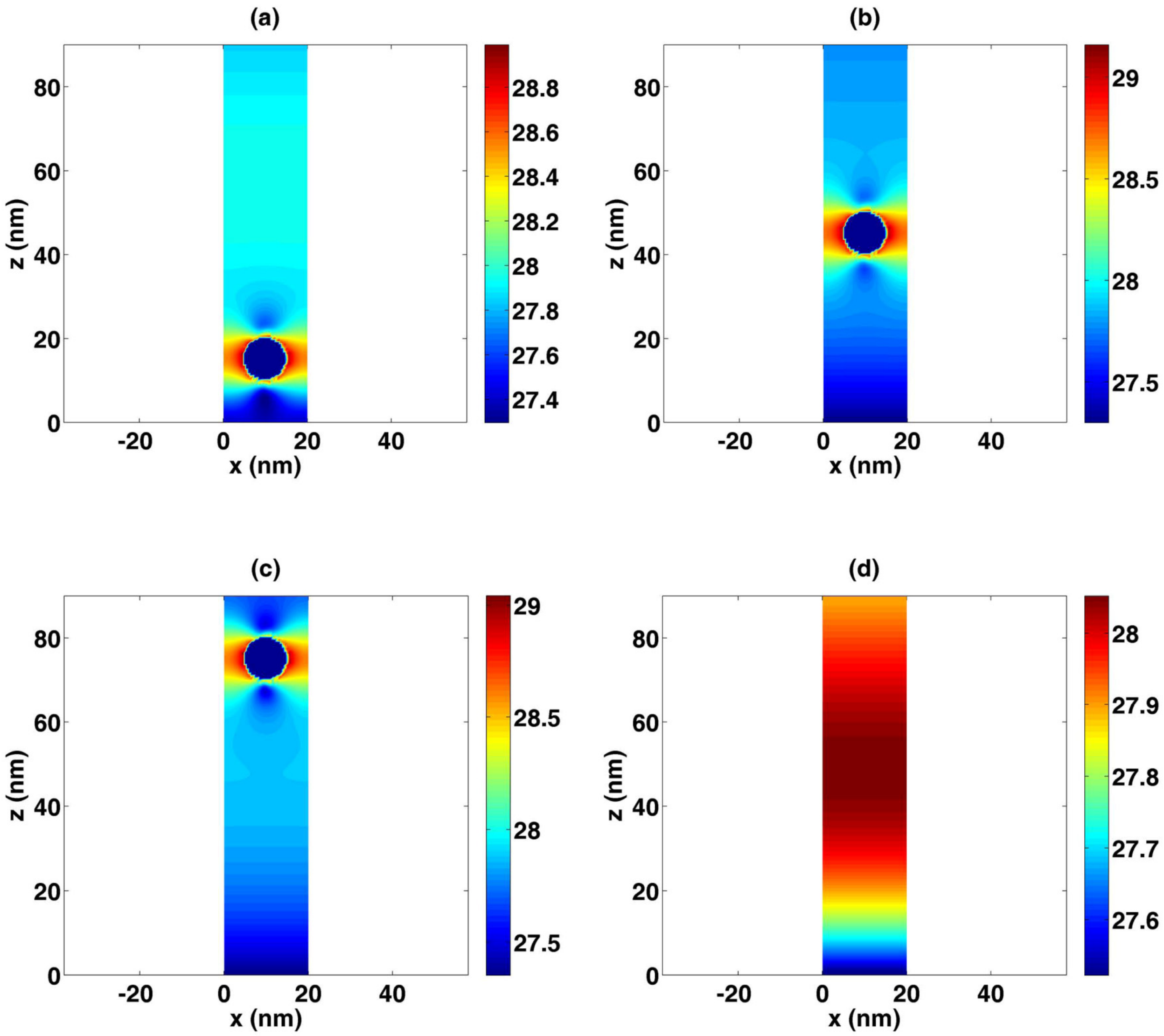

Figure S2. Spatial distribution of exciton generation at the active layer of small molecule solar cells. Metallic nanoparticles are embedded at the different regions of active layer. (a) near-cathode case; (b) middle case; (c) near-anode case; (d) standard cell without nanoparticles. The colorbars show the amplitudes of exciton generation ($m^{-3}s^{-1}$) with a logarithmic scale. A strong plasmonic enhancement can be observed around the nanoparticles. The exciton generation shown is the average value of those for TE and TM polarizations.

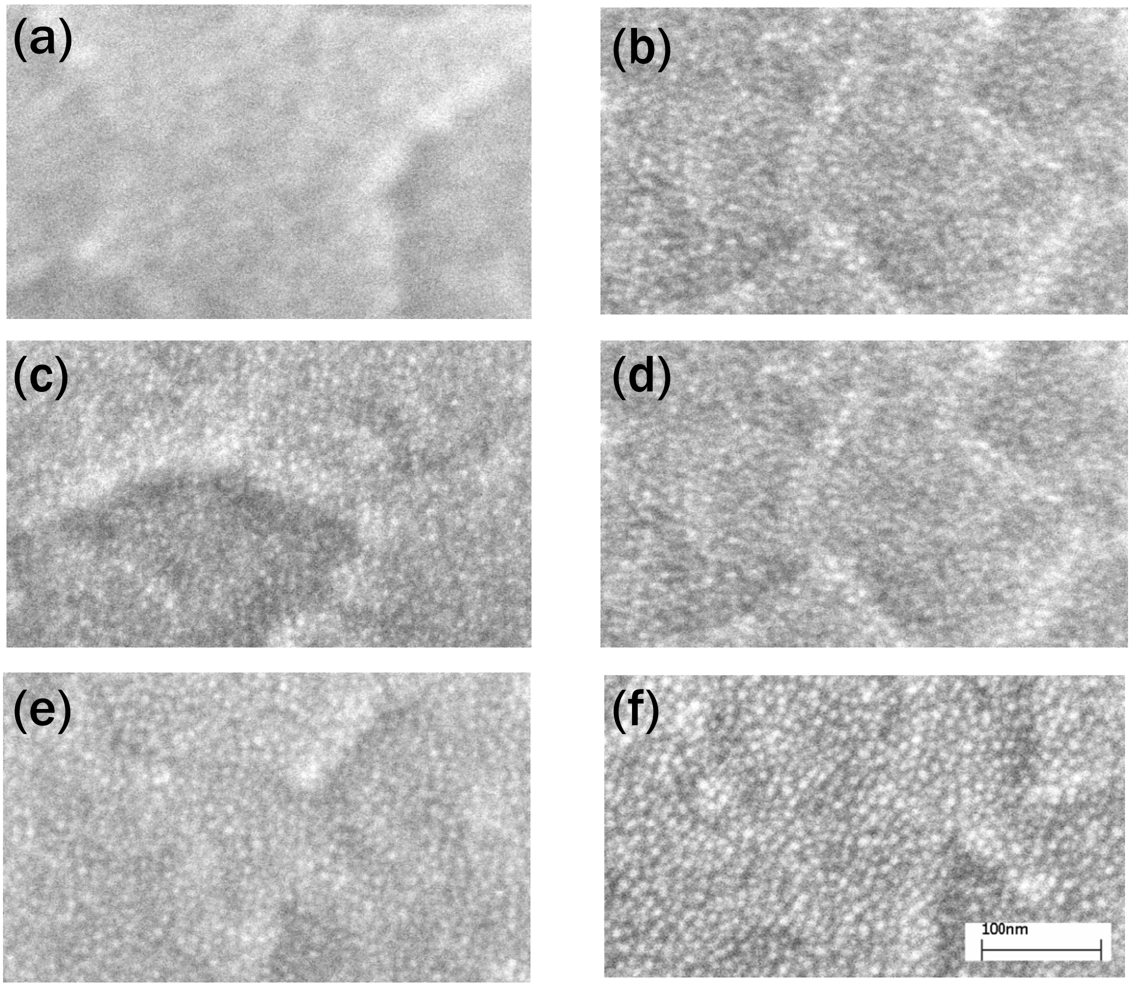

Figure S3. Top-viewed SEM images of the blend CuPc:C_{60} thin film (a) without thermally evaporated silver film, (b) with evaporated silver film of 0.5 nm, (c) 1.0 nm, (d) 1.5 nm, (e) 2 nm, (f) 3 nm. All the scales are the same as (f). Various sized silver NPs were formed after the thermal evaporation procedure.

Electron and hole mobility measurement

Electron and hole mobility for the active material of CuPc:C_{60} (1:1 weight ratio) are measured by the space charge limited current (SCLC) technique [*Jpn. J. Appl. Phys. 41, 5626, 2002*] with device structures of ITO/ Ca (4 nm)/ CuPc:C_{60} (100 nm)/ LiF (1 nm)/ Al (120 nm) and ITO/ PEDOT:PSS (30 nm)/ CuPc:C_{60} (100 nm)/ MoO_3 (10 nm)/ Al (120 nm), respectively. The electron and hole mobility are 1.0×10^{-2} $cm^2/(V\cdot s)$ and 3.4×10^{-7} $cm^2/(V\cdot s)$, which are very unbalanced with each other.

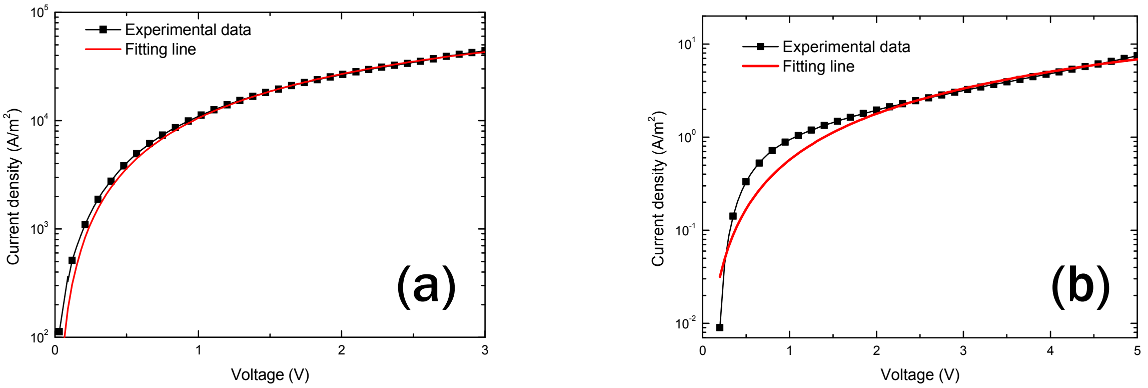

Figure S4. The electron and hole mobility for the active material of CuPc:C_{60} (1:1 weight ratio) measured by the SCLC technique. (a) electron mobility; (b) hole mobility.

Supplementary Note 2: manipulate photocarrier transport path in amorphous silicon solar cells. Device structure is TCO anode/ p^+ silicon (10 nm)/ intrinsic silicon (460 nm)/ n^+ silicon (30 nm)/metallic cathode. The total active layer thickness is L=500 nm. Governing equations and simulation parameters are taken from literature works [*Journal of Applied Physics, 53(1), 712-719, Jan. 1982; Optics Express, 19 (S4), A888-A896, Jun. 2011*]. Most photocarriers (electrons and holes) are generated at the intrinsic silicon layer. Particularly, photocarrier mobility of amorphous silicon is higher than that of organic materials; and is balanced with each other. For intrinsic silicon material, electron and hole mobility are, respectively, 4.6×10^{-2} $cm^2/(V\cdot s)$ and 9.2×10^{-3} $cm^2/(V\cdot s)$. To understand the role of spatial distributions of exciton generation in affecting device performance, spatial distributions of exciton generation in the active layer are set as

$$G_{pl}^{m}(z)=\begin{cases} 0, & 0\le z<2L/5 \\ 5\bar{G}_p, & 2L/5\le z<3L/5 \\ 0, & 3L/5\le z<L \end{cases} \tag{S1}$$

$$G_{pl}^{a}(z)=\begin{cases} 0, & 0\le z<4L/5 \\ 5\bar{G}_p, & 4L/5\le z<L \end{cases} \tag{S2}$$

$$G_{pl}^{c}(z)=\begin{cases} 5\bar{G}_p, & 0\le z<L/5 \\ 0, & L/5\le z<L \end{cases} \tag{S3}$$

$$G_p(z)=\left\{\bar{G}_p, \quad 0\le z<L\right. \tag{S4}$$

where G_{pl}^{m}, G_{pl}^{a}, and G_{pl}^{c} are the exciton generation rates with respect to the three solar cells (middle, near-anode, and near-cathode cases) as depicted in **Figures 1(a-c)** of manuscript. The cathode and anode are located at z=0 and z=L, respectively. For an intuitive comparison, exciton generation of the standard cell is assumed to be uniform at the active layer [Eq. (S4)]. It is worthy of noting that all the devices have the same averaged exciton generation rate $\bar{G}_p$ (with any optical absorption enhancements).

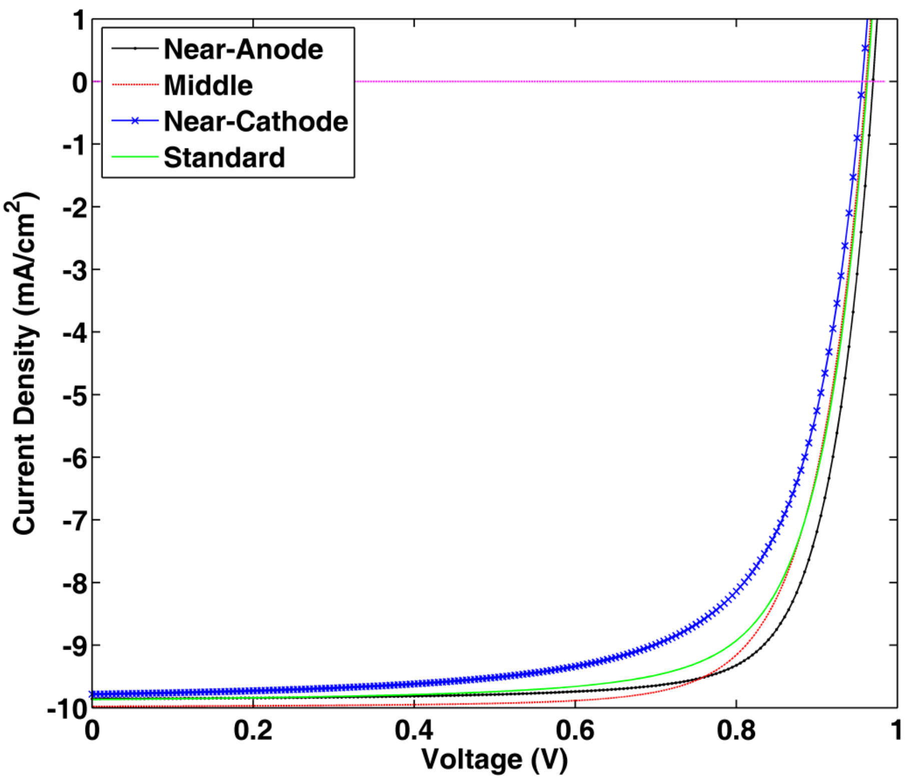

Figure S5. Theoretical *J*-*V* responses for amorphous silicon solar cells with different exciton generation distributions. Most photocarriers (electrons and holes) are generated at the intrinsic silicon layer with electron mobility of 4.6×10^{-2} $cm^2/(V\cdot s)$ and hole mobility of 9.2×10^{-3} $cm^2/(V\cdot s)$.

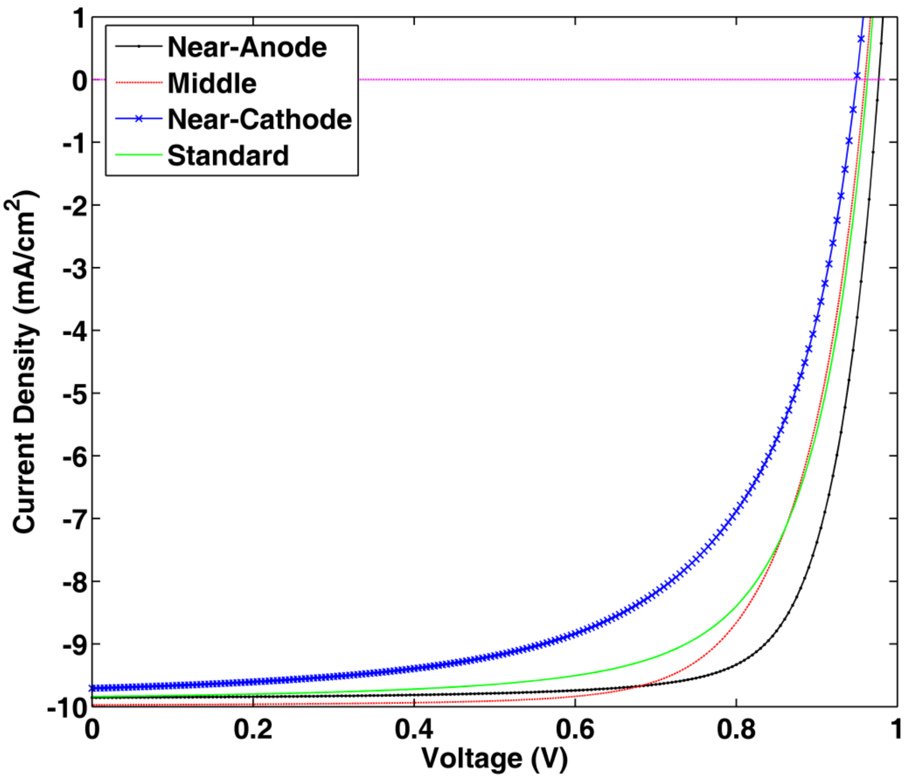

Figure S6. Theoretical *J*-*V* responses for amorphous silicon solar cells with different exciton generation distributions. Most photocarriers (electrons and holes) are generated at the intrinsic silicon layer with electron mobility of 4.6×10^{-2} $cm^2/(V\cdot s)$ and hole mobility of 4.6×10^{-3} $cm^2/(V\cdot s)$.

Figure S5 shows the *J-V* responses for amorphous silicon solar cells with different exciton generation distributions described by Eqs. (S1-S4). **Table S1** lists corresponding characteristic parameters including short-circuit current (J_{sc}), open-circuit voltage (V_{oc}), fill factor (FF), and power conversion efficiency (PCE). The near-anode case, which shortens transport paths of low-mobility holes, achieves the highest PCE with the maximum FF and V_{oc}. Due to a significantly reduced built-in potential, bulk recombination of photocarriers at the maximum power point dominates device performance of amorphous silicon solar cells. To further confirm the importance and significance of optical absorption redistribution at the intrinsic silicon layer, we reduce hole mobility half and keep electron mobility unchanged, i.e. μ_e=4.6×10^{-2} cm^2/(V·s) and μ_h=4.6×10^{-3} cm^2/(V·s). Exciton generations are still set by Eqs. (S1-S4). **Figure S6 and Table S2** show the changed *J-V* responses and characteristic parameters. Interestingly, the near-anode case shows more significant advantages over other cases in terms of $V_{oc,}$ FF, and PCE. Regarding the near-anode case (exciton generation is nonzero from 400 nm to 500 nm), the electron transport length (L_e) and hole transport length (L_h) are approximate to be 450 nm and 50 nm. Hence, L_e/L_h=9 is close to μ_e/μ_h=10, which agrees well with the general design rule in Eq. (2) of manuscript. This is the reason why the PCE of the near-anode case with a low hole mobility can be even higher than that of the near-anode case with a high hole mobility (See **Table S1** and **Table S2**). Consequently, the general design rule manipulating photocarrier transport path and thus bulk recombination is indeed useful to design high-performance thin-film photovoltaics.

Table S1. Characteristic parameters for amorphous silicon solar cells with different exciton generation distributions. Most photocarriers (electrons and holes) are generated at the intrinsic silicon layer with electron mobility of 4.6×10^{-2} cm^2/(V·s) and hole mobility of 9.2×10^{-3} cm^2/(V·s).

Exciton Distribution	J_{sc} (mA/cm^2)	V_{oc} (V)	FF	PCE (%)
Standard (uniform)	98.669	0.960	0.754	7.143
Middle	99.770	0.960	0.765	7.326
Near-Cathode	97.830	0.955	0.700	6.542
Near-Anode	98.563	0.970	0.786	7.511

Table S2. Characteristic parameters for amorphous silicon solar cells with different exciton generation distributions. Most photocarriers (electrons and holes) are generated at the intrinsic silicon layer with electron mobility of 4.6×10^{-2} cm^2/(V·s) and hole mobility of 4.6×10^{-3} cm^2/(V·s).

Exciton Distribution	J_{sc} (mA/cm^2)	V_{oc} (V)	FF	PCE (%)
Standard (uniform)	98.392	0.960	0.713	6.737
Middle	99.720	0.960	0.730	6.991
Near-Cathode	97.067	0.945	0.627	5.754
Near-Anode	98.546	0.975	0.784	7.528
